# A Low-Profile Antenna for On-Body and Off-Body Applications in the Lower and Upper ISM and WLAN Bands

**DOI:** 10.3390/s23020709

**Published:** 2023-01-08

**Authors:** Esraa Mousa Ali, Wahaj Abbas Awan, Syeda Iffat Naqvi, Mohammed S. Alzaidi, Abdullah Alzahrani, Dalia H. Elkamchouchi, Francisco Falcone, Turki E. A. Alharbi

**Affiliations:** 1 Faculty of Aviation Sciences, Amman Arab University, Amman 11953, Jordan; 2 Department of Information and Communication Engineering, Chungbuk National University, Cheongju 28644, Republic of Korea; 3 Telecommunication Engineering Department, University of Engineering and Technology, Taxila (UET Taxila), Taxila 47050, Pakistan; 4 Department of Electrical Engineering, College of Engineering, Taif University, P.O. Box 11099, Taif 21944, Saudi Arabia; 5 Department of Information Technology, College of Computer and Information Sciences, Princess Nourah bint Abdulrahman University, P.O. Box 84428, Riyadh 11671, Saudi Arabia; 6 Electrical Engineering and Communications Department, Campus Arrosadía, Universidad Pública de Navarra, E-31006 Pamplona, Spain; 7 Institute of Smart Cities, Campus Arrosadía, Universidad Pública de Navarra, E-31006 Pamplona, Spain; 8 Tecnologico de Monterrey, School of Engineering and Sciences, Monterrey 64849, Mexico

**Keywords:** dual-band, compact antenna, ISM, WLAN, Wi-Fi

## Abstract

The article presents a Co-planar Waveguide (CPW) fed antenna of a low-profile, simple geometry, and compact size operating at the dual band for ISM and WLAN applications for 5G communication devices. The antenna has a small size of 30 mm × 18 mm × 0.79 mm and is realized using Rogers RT/Duroid 5880 substrate. The proposed dual-band antenna contains a CPW feedline along with the triangular patch. Later on, various stubs are loaded to obtain optimal results. The proposed antenna offers a dual band at 2.4 and 5.4 GHz while covering the impedance bandwidths of 2.25–2.8 GHz for ISM and 5.45–5.65 GHz for WLAN applications, respectively. The proposed antenna design is studied and analyzed using the Electromagnetic (EM) High-Frequency Structure Simulator (HFSSv9) tool, and a hardware prototype is fabricated to verify the simulated results. As the antenna is intended for on-body applications, therefore, Specific Absorption Rate (SAR) analysis is carried out to investigate the Electromagnetic effects of the antenna on the human body. Moreover, a comparison between the proposed dual-band antenna and other relevant works in the literature is presented. The results and comparison of the proposed work with other literary works validate that the proposed dual-band antenna is suitable for future 5G devices working in Industrial, Scientific, Medical (ISM), and Wireless Local Area Network (WLAN) bands.

## 1. Introduction

Rapid development in wireless communication technology for 5G deployment required compact, smart, lightweight, efficient, and low-cost devices. Due to the modifications in wireless communication devices, improvement in antenna design is also required [1]. The compact, multiband, geometrically simple, and low-profile antennas are the required candidates for current and future wireless communication systems [2].

Several wireless applications, predominantly health care, connectivity, Bluetooth, Wi-Fi, and Global Positing System (GPS), are operating on Industrial, Scientific, Medical (ISM), and Wireless Local Area Network (WLAN) bands [3]. The antennas offering ISM and WLAN dual-bands are considered excellent candidates for wireless devices [4]. Researchers have recently proposed several antennas operating on the ISM and WLAN bands [5,6,7,8,9,10,11,12,13,14,15,16,17,18,19,20,21,22]. The reported works [5,6,7,8,9,10,11] demonstrate various antenna designs operating over the 2.4 GHz ISM band. The antennas proposed in [5,6] have simple geometry and offer a high gain of 8.68 dBi and 8.5 dBi, respectively. However, the proposed designs have comparatively larger dimensions of 60 mm × 80 mm × 3 mm and 100 mm × 15 mm × 3 mm, respectively. In [7], the compact and wideband antenna for ISM applications is presented. This antenna has low radiation efficiency and complex geometry. In [8,9], geometrically simple and compact antennas are reported. These antennas, however, attained low gain values of 1.7 dBi and 1 dBi, respectively. Likewise, antennas with moderate gain and compact size are reported in [10,11]. However, the drawback associated with these antennas is complex geometry and narrow bandwidth of 0.5 GHz and 0.12 GHz, respectively.

For 5.2 GHz WLAN applications, various works have been reported in the literature [12,13,14,15,16,17]. In [12], a geometrically simple E-shaped patch antenna is reported to operate at 5.1–5.32 GHz. The antenna proposed in [13] has large dimensions of 142 mm × 60 mm × 0.5 mm and complex geometry, as well as narrow bandwidth compared to the required bandwidth for 5.2 GHz WLAN applications. A pie-shaped slot antenna for 5.2 GHz is presented in [14]. The antenna has a compact size of 16 mm × 19 mm × 1.6 mm but offers narrow bandwidth of 0.05 GHz. On the other hand, high-gain antennas offering gains of 6.2 dBi and 3.3 dBi are reported in [15,17]. Although the reported antennas have high gain, complex geometries and large antennas are observed.

Furthermore, tri-band antennas operating at 2.4 GHz, 3.3 GHz, and 5.2 GHz bands are demonstrated in [18,19,20,21,22]. The antenna presented in [18] has novel geometry and has a size of 42 mm × 36 mm × 2.4 mm. However, the design has structural complexity due to the multilayer configuration. In [19], a compact antenna is proposed. The bandwidth obtained by this antenna at the operational bands is narrow, ranging from 2.4–2.48/5.15–5.35 GHz. Likewise, an antenna with a peak gain of 4.8/4.7 dB at 2.4/5.8 GHz is demonstrated in [20]. Although the radiator has a high gain, the employment of an Artificial Magnetic Conductor (AMC) increased the geometrical complexity of the antenna. In [21,22], dual-band antennas for ISM and WLAN applications are presented. The antennas offer 2.4–2.6/4.9–5.3 GHz and 2.34–2.5/5.06–5.9 GHz bandwidth, respectively. The setback of these antennas is the large dimensions of 100 mm × 100 mm × 0.8 mm and 74 mm × 27 mm × 17 mm, respectively.

Considering the inadequacies of the previously reported works, a dual-band low-profile antenna having a simple and compact geometrical configuration is proposed in this work for ISM and WLAN bands. The dual-band is achieved by the insertion of various stubs into radiating patch antenna. The proposed antenna obtained high gain, large operational bandwidth, and good radiation efficiency, which ascertains the antenna’s suitability for on- and off-body applications. The rest of the paper is divided into three sections. The design methodology, parametric analysis of key parameters, and proposed design with optimized parameters are discussed in Section 2. The hardware prototype, measured results, and comparative analysis of simulated and measured results, as well as SAR analysis, are provided in Section 3. Section 3 also contains the comparison table, which compares the proposed antenna with state-of-the-art work published in the literature operating on the same frequency bands. The work is concluded in Section 4, along with references.

## 2. Design of Proposed Dual-Band CPW-Fed Antenna

### 2.1. Geometry of Antenna

**Figure 1:**
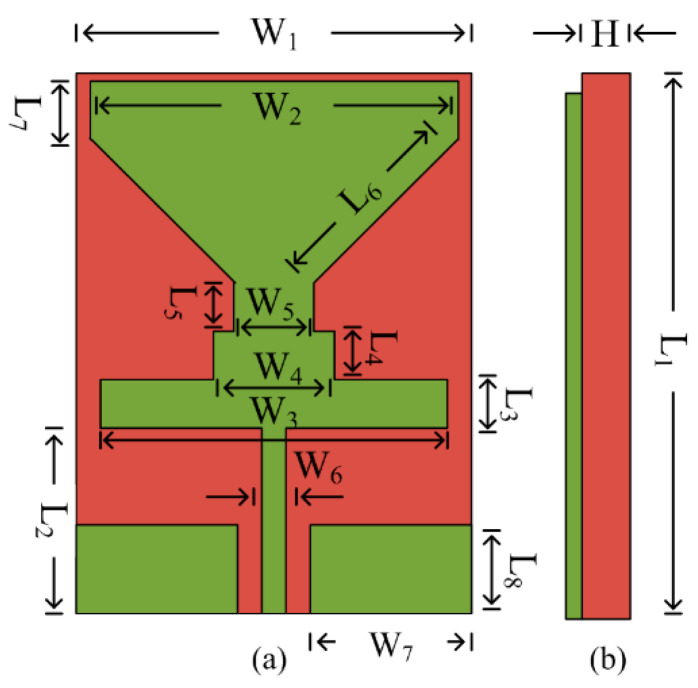
Geometrical configuration of proposed compact CPW-fed antenna Operating on ISM and WLAN applications (**a**) top-view (**b**) side-view.

Figure 1 depicts the geometry of the proposed CPW-fed antenna resonating at two bands, i.e., 2.5 and 5.4 GHz. The antenna is modeled on Rogers RT/5880, with dielectric loss and permittivity of 0.0012 and 2.2 and thickness of 0.79 mm. The proposed antenna has an overall compact size of L_1_ × W_1_ × H = 30 mm × 18 mm × 0.79 mm. The antenna geometry consists of a triangular radiator loaded with rectangular stubs. The stubs are inserted into the radiator to improve return loss and make the antenna operational on the dual band. The optimized parameter of the antenna is given below.

L_1_ = 30, L_2_ = 11, L_3_ = 2, L_4_ = 3, L_5_ = 3, L_6_ = 9.2, L_7_ = 3.5, L_8_ = 4, W_1_ = 18, W_2_ = 17, W_3_ = 16, W_4_ = 6, W_5_ = 4, W_6_ = 2, H = 0.79. (All units are in millimeters).

### 2.2. Design Stages of Proposed Antenna

**Figure 2:**
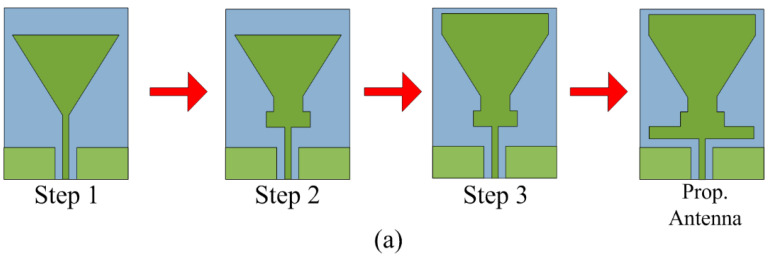

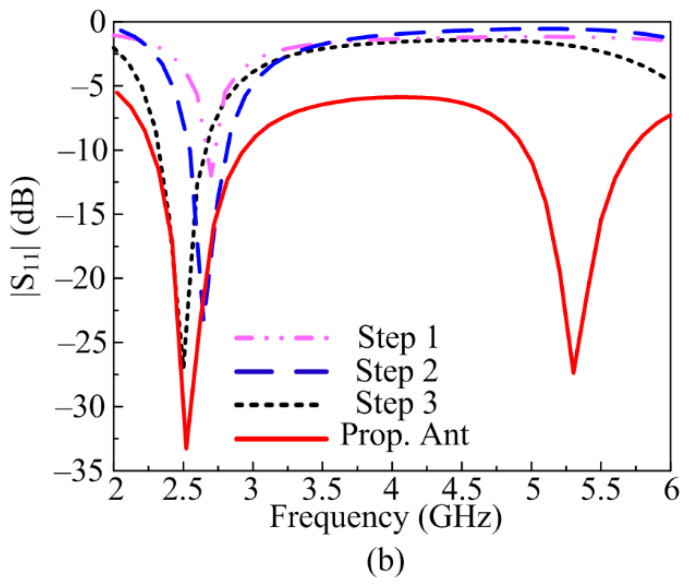
Design stages of the proposed antenna and its impact on the S-parameter. (**a**) The proposed dual-band antenna is obtained after carrying out various design steps. (**b**) The corresponding S-parameter results for each design stage.

The proposed dual-band antenna is obtained after carrying out various design steps, as given in Figure 2a, and the corresponding S-parameter results for each design stage are given in Figure 2b. Initially, a triangular patch antenna with a microstrip feedline is designed, which offers resonance at 2.6 GHz. In the second stage, the two rectangular stubs are inserted between the radiating triangular patch and feedline. This stage slightly shifts the resonant band toward the lower frequencies, and a downward shift is also observed, as depicted in Figure 2b. In the third stage, a rectangular stub is loaded on top of the triangular patch, which further improves the return loss and shifts resonance to 2.4 GHz. In the last stage, a rectangular stub is added below the existing stubs, as shown in Figure 2a. Due to this modification in antenna design, another resonance at 5.2 GHz is obtained, which makes the proposed antenna operational on a dual-band.

### 2.3. Parametric Analysis of Key Parameters

#### 2.3.1. Lower Stub Responsible for 5.2 GHz Band

**Figure 3:**
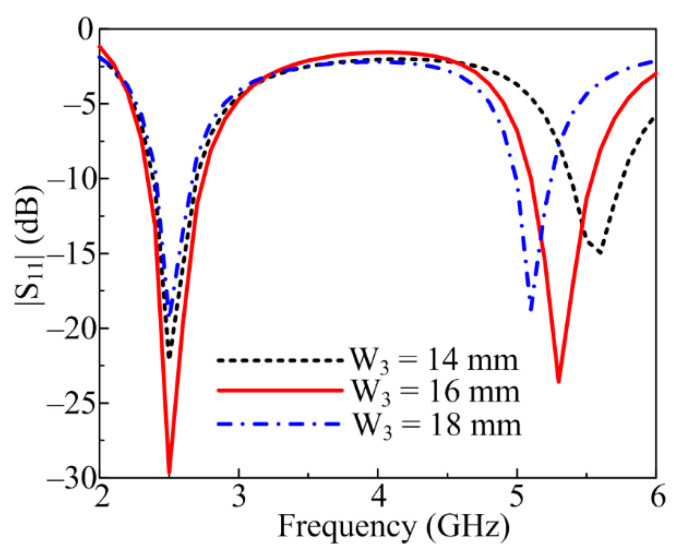
Parametric analysis of proposed antenna by varying lower stub (W_3_).

Section 2.2 demonstrates design evolution and clearly shows that various stubs inserted play a key role in antenna performance. The lower stub (W_3_) is greatly responsible for obtaining the second antenna resonance at a higher band of 5.2 GHz. At an optimal value of W_3_ = 16 mm, the antenna resonates for the 5.4 GHz band ranging from 5–5.75 GHz. If the W_3_ value is increased to 18 mm, the antenna bandwidth is compromised, as shown in Figure 3. On the other hand, if W_3_ is fixed to 14 mm, the second band shifts toward the right side, ranging from 5.4–5.85 GHz.

#### 2.3.2. Length of the Radiator Responsible for 2.45 GHz Band

**Figure 4:**
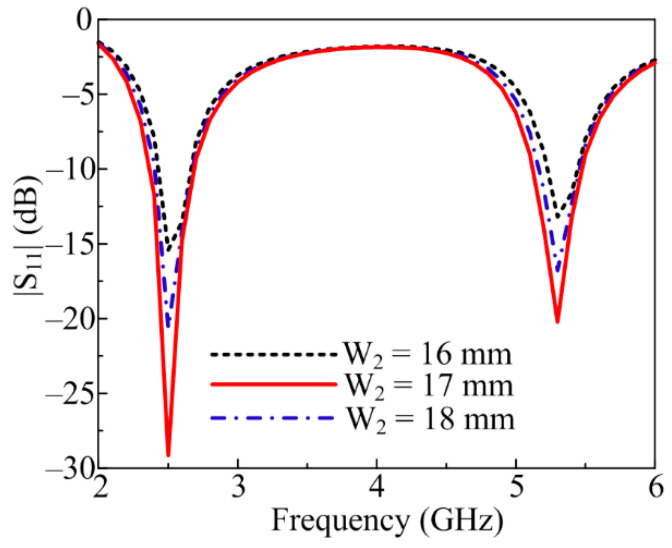
Parametric analysis of proposed antenna by varying upper stub (W_2_).

The rectangular stub at the top side of the triangular patch antenna also plays a vital role in antenna radiation characteristics. By varying the W_2_, upward and downward shifting of the resonating bands is observed. When the value of W_2_ is set to 17 mm, the antenna shows optimal S-parameter results, as depicted in Figure 4. However, when the value of W_2_ is changed to 16 and 17 mm, an upward shift of the two resonating bands is noticed, as shown in Figure 4.

### 2.4. Numerical Analysis

**Figure 5:**
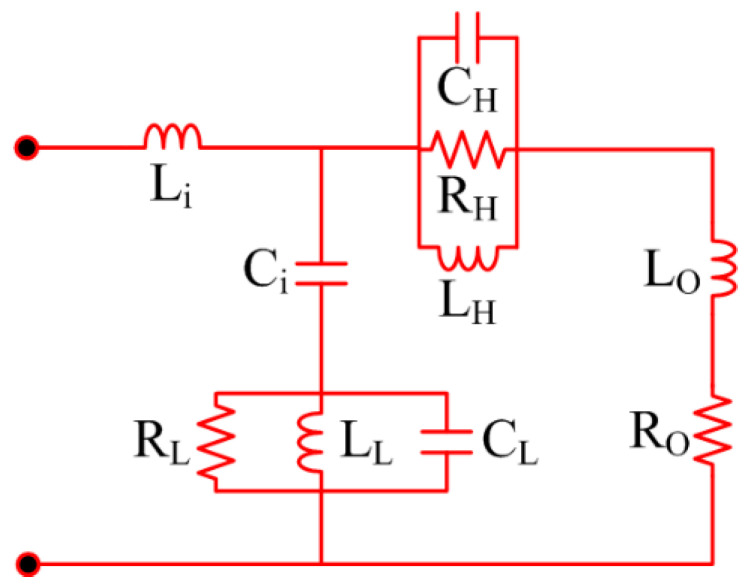
Equivalent circuit model of proposed dual-band antenna (C_i_ = 2.8 pF; L_i_ = 6 nH; R_L_ = 760 Ω; L_L_ = 2; C_L_ = 1.1 pF; C_H_ = 3 pF; R_H_ = 1500 Ω; L_H_ =4.3 nH; L_O_ = 2 nH; R_O_ = 15 Ω).

**Figure 6:**
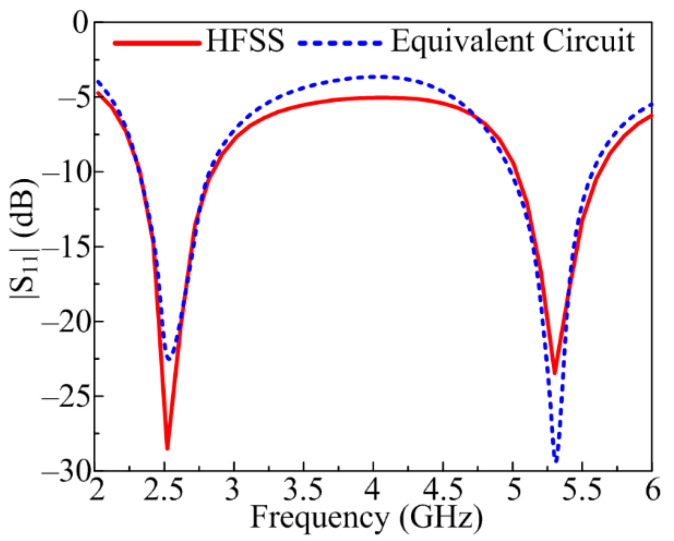
|S_11_| comparison of antenna designed using HFSS and equivalent circuit model.

Figure 5 exhibits the equivalent circuit model for the proposed antenna that is suggested for on- and off-body communication. As shown in Figure 5, the model circuit comprises of pair of parallel resistor–inductor–capacitor (RLC) circuits with three capacitors, three resistors, and four inductors. The |S_11_| of the model circuit may be altered by tuning the values of the resistors, capacitors, and inductors. The RLC circuit, connected with one inductor and a capacitor on the left side of the circuit, generates the lower frequency band of 2.4 GHz. Similarly, the RLC circuit connected with an inductor and resistor on the right side of the circuit is responsible for generating the higher frequency band of 5.2 GHz. Figure 6 illustrates that the S-parameter results of the proposed antenna obtained through HFSS are in close agreement with the one obtained in the case of the equivalent circuit of the proposed antenna.

## 3. Measured and Simulated Results of the Proposed Antenna

### 3.1. Measurement Setup

**Figure 7:**
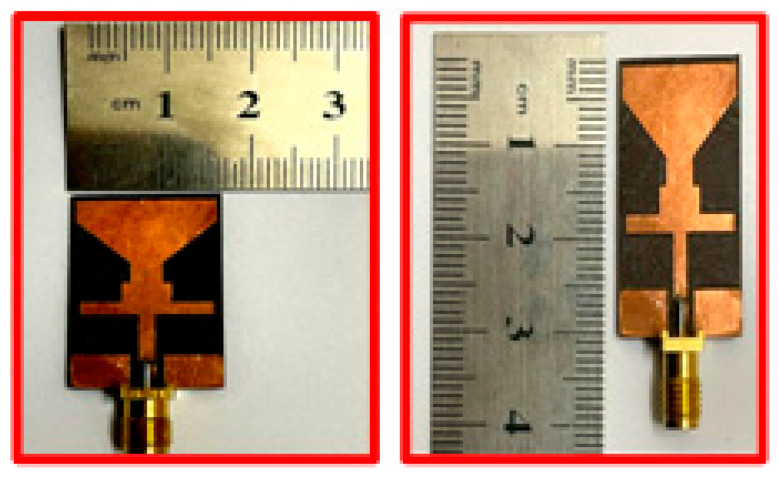
Hardware Prototype of proposed dual-band antenna.

The fabrication of the proposed antenna is carried out to verify the simulated results. Figure 7 illustrates the prototype of the proposed antenna. The SMA female jack Edge used has four legs [23]. End-launch Printed Circuit Board (PCB) connectors are used to excite the proposed dual-band antenna. A Vector Network Analyzer (VNA) with the model number HP 8720D ranging from 0.05–13.5 GHz is utilized to verify the antenna’s S-parameters. To examine and verify the far-field results, a broadband horn antenna EMCO Type 3115 having a bandwidth range from 1–18 GHz is used. The testing and reference antenna has a distance of 3 m, according to the standard for verifying the results.

### 3.2. Scattering Parameter

**Figure 8:**
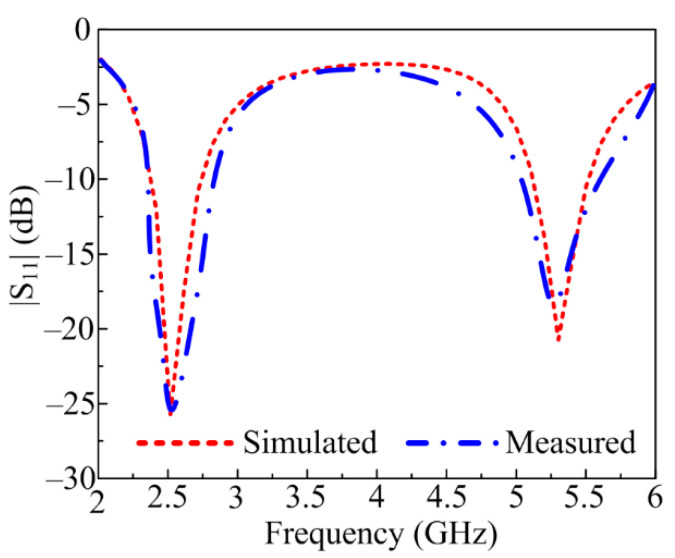
Comparison between simulated and measured S-parameter of proposed work.

The comparison of simulated and measured S-parameters for the proposed dual-band antenna is given in Figure 8. The measured |S_11_| plots of the proposed antenna demonstrate a dual-band at 2.45 GHz and 5.4 GHz with bandwidth ranging from 2.25–2.8 GHz and 5–5.65 GHz, respectively. Moreover, good concurrency between simulated and measured results is noticed. The promising radiation characteristics of the proposed antenna make it a possible nominee for future 5G communication devices in the ISM band operating at 2.4 GHz and the WLAN band operating at 5.4 GHz.

### 3.3. Measured and Simulated Gain

**Figure 9:**
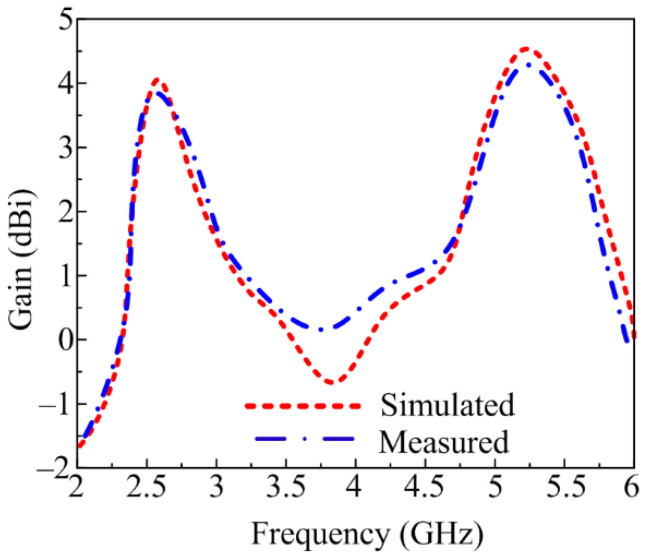
Comparison between measured and simulated gain of suggested antenna.

The simulated and measured gain of the CPW-fed dual-band antenna is given in Figure 9. The figure shows that the proposed antenna offers a gain >3.7 dBi for the 2.25–2.8 GHz ISM band and a gain >4.3 dBi for the 5–5.65 GHz WLAN band. At operational frequencies of 2.45 GHz and 5.4 GHz, the antenna provides peak gains of 3.9 dBi and 4.8 dBi, respectively.

### 3.4. Simulated Radiation Efficiency

**Figure 10:**
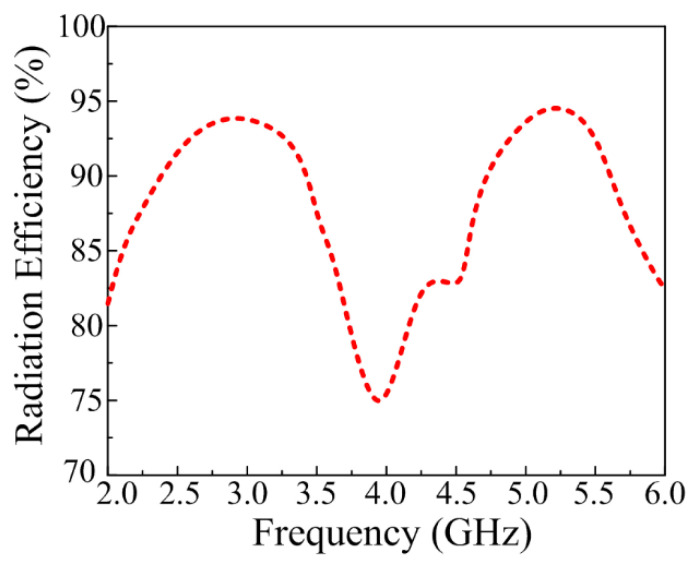
Estimated radiation efficiency of the proposed work.

Figure 10 expresses the numerically estimated radiation efficiency of the optimized CPW-fed dual-band antenna. The proposed work offers efficiency >92% at bandwidth ranging from 2.25–2.8 GHz and 5–5.8 GHz. The plot shows that the antenna offers a peak radiation efficiency of 94% at 2.45 GHz and 95% at 5.4 GHz.

### 3.5. Measured and Simulated Radiation Pattern

**Figure 11:**
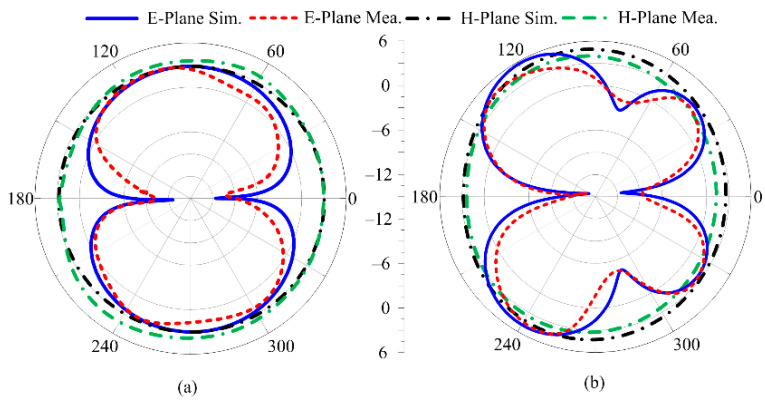
Simulated and measured radiation pattern of prosed dual-band antenna at (**a**) 2.4 GHz (**b**) 5.2 GHz.

Figure 11 illustrates the proposed antenna’s simulated and measured radiation patterns at 2.45 GHz and 5.4 GHz. It is shown that a dual-band antenna for 2.45 GHz gives an omnidirectional radiation pattern at the main H-plane and a bi-directional radiation pattern at the main E-plane. In contrast, the antenna for 5.4 GHz gives an omnidirectional radiation pattern at the primary H-plane and a butterfly-shaped radiation pattern at the primary E-plane. Due to multiple stub insertion to achieve a dual band, the radiation pattern at 5.4 GHz has been altered.

### 3.6. SAR Analysis

The antennas for on-body applications work very close to the human body, thus receiving and radiating electromagnetic waves (EM). EM radiations are harmful if human tissue absorbs excess amounts, producing ionization and heating effects. In order to ascertain human safety, the amount of exposure of human tissues to EM radiation is standardized by the Institute of Electrical and Electronics Engineers (IEEE), the International Commission on Non-Ionizing Radiation Protection (ICNIRP), and the Federal Communication Commission (FCC). The amount of electromagnetic energy absorbed by human tissue is measured by the Specific Absorption Rate (SAR). It is calculated by averaging across a volume of either 1 g or 10 g. In Europe, the SAR limit is 2 W/kg for 10 g of tissue, whereas the United States has a limit of 1.6 W/kg for 1 g of tissue. Equation (1) can be utilized to assess the SAR values [23].

(1) $$\mathrm{SAR}=\left|E^{2}\right|\frac{\sigma }{\rho }$$

where E represents electric field intensity (V/m), σ is electrical conductivity (S/m), and ρ stands for mass density (kg/m^3^), respectively.

**Figure 12:**
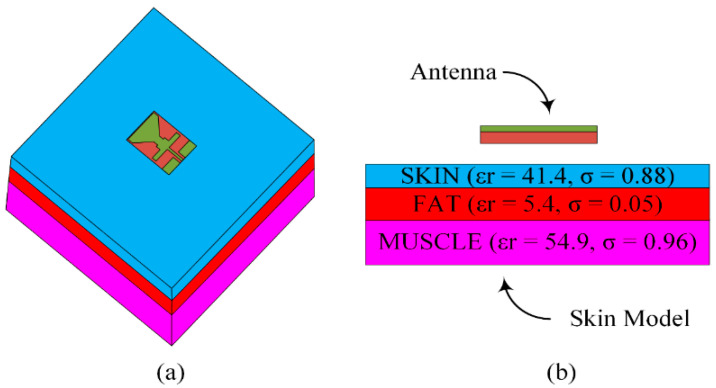
Simulation setup for SAR analysis having an antenna with 3-layered human body model (**a**) perspective view (**b**) side view.

**Figure 13:**
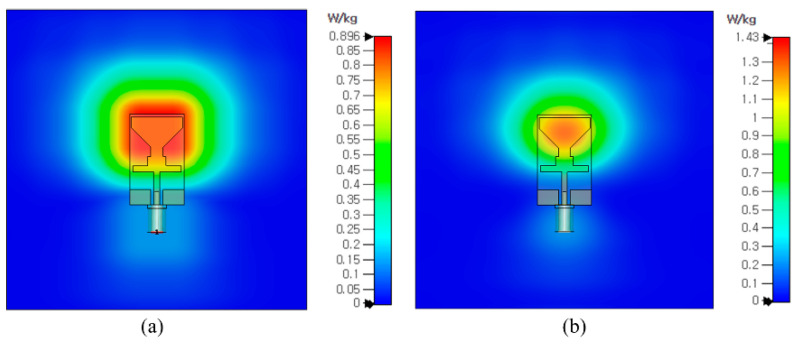
SAR of prosed dual-band antenna at (**a**) 2.4 GHz (**b**) 5.2 GHz.

Figure 12 shows the simulation setup for the SAR analysis. The antenna is placed on top of a three-layered human-body-equivalent model having a total size of 100 mm × 100 mm. The standard thicknesses of skin (2 mm), fat (3 mm), and muscle (8 mm) were chosen and assigned the respective dielectric constant and thermal conductivity, as shown in Figure 12b. The space between the skin model and the antenna is 2 mm. The simulated SAR value for the proposed antenna at 2.4 GHz is 0.896 W/kg, and at 5.2 GHz, it is 1.43 W/kg for 1 g of tissue, both of which are within the allowable limits of 1.6 W/kg for 1 g of tissue (as depicted in Figure 13). The SAR values of the proposed antenna are within the permissible range with an input power of 0.2 W. Therefore, the suitability of this antenna for the on-body applications operating at ISM and WLAN frequency bands is validated.

### 3.7. Comparison of Proposed Work with the Literature

The comparison of the proposed CPW-fed dual-band compact antenna with already published work in the literature is summarized in Table 1. It is observed that the reported works either have large sizes or complex geometries as compared to the antenna proposed in this work or the one with a narrow bandwidth or low gain. It is also noted that most reported antennas have a single operational band or low-radiation efficiency. In addition, for the earlier reported antennas, SAR analysis has not been conducted. These discrepancies of the works presented previously in the literature verify that the dual-band antenna proposed in this work is suitable for on-body and off-body smart communication devices operating at ISM and WLAN bands due to its promising features such as simple and compact geometry, significant gain, high radiation efficiency, and SAR values within the acceptable range.

## 4. Conclusions

This paper has presented an antenna resonating over 2.4 GHz and 5.4 GHz. The CPW-fed antenna with a triangular-shaped patch was initially modeled for ISM applications, which resonates at 2.4 GHz. Later, various stubs were loaded to improve the return loss and to obtain another band at 5.4 GHz. The antenna has a compact size, simple geometrical configuration, and low profile. The proposed CPW-fed dual-band antenna offers 2.25–2.8 GHz and 5–5.65 GHz for ISM and WLAN applications, respectively. The peak gain of 3.9 dBi with a radiation efficiency of 92% is attained at 2.45 GHz, whereas a peak gain of 4.8 dBi with a radiation efficiency of 94% is obtained at 5.4 GHz by the proposed work. In addition, SAR analysis ascertained that the proposed antenna is safe to be used in close proximity to the human body. Moreover, the results and comparison with state-of-the-art work show that the proposed work is suitable for current and future 5G portable on-body and off-body devices working over dual-band applications of ISM and WLAN.

## Figures and Tables

**Table 1:** Comparison of proposed dual-band antenna with the previously presented antennas in the literature operating at same bands.

Ref	Size (mm^3^)	Operational Mode	Resonant Frequency (GHz)	Operational Bandwidth (GHz)	Gain (dBi)	Radiation Efficiency (%)
[5]	60 × 80 × 3	Single	2.42	2.37–2.44	8.68	-
[6]	200 × 15 × 3	Single	2.3	2.2–2.57	8.5	-
[7]	45 × 42 × 1.27	Single	2.7	2.38–3.1	4.8	-
[8]	40 × 10 × 1.6	Single	2.4	2.38–2.43	1.34	79
[9]	38 × 33.2 × 1.5	Single	2.4	2.37–2.43	1	-
[10]	40 × 30 × 1.6	Single	2.4	2.2–2.73	3.28	-
[11]	76 × 57.2 × 1.6	Single	2.4	2.38–2.42	3.1	-
[12]	30 × 25 × 3.2	Single	5.2	5.1–5.32	6.3	-
[13]	142 × 60 × 0.5	Single	5.2	5.17–5.26	4.5	-
[14]	16 × 19 × 1.6	Single	5.38	5.35–5.4	5.83	-
[15]	55 × 52 × 1.57	Single	5.2	5.1–5.3	6.2	-
[16]	32 × 28 × 1.6	Dual	2.4/5.2	2.3–2.5/4.9–6	-	-
[17]	14.5 × 14 × 1.6	Single	5.2	5–5.3	3.3	41.7
[18]	42 × 36 × 2.4	Dual	2.4/3.3	2.2–2.45/3.3–3.45	4.6/4.2	-
[19]	18 × 17 × 4	Dual	2.4/5.2	2.4–2.48/5.15–5.35	4.1/1.4	-
[20]	44.5 × 44.5 ×1.6	Dual	2.4/5.8	2.2–2.4/5.7–5.9	4.8/4.7	-
[21]	100 × 100 × 0.8	Dual	2.3/5.2	2.4–2.6/4.9–5.3	4.8/5.7	70/70
[22]	74 × 27 × 17	Dual	2.4/5.4	2.34–2.5/5.06–5.9	3.8/6.8	-
**This Work**	**30 × 18 × 0.79**	**Dual**	**2.4/5.4**	**2.25–2.8/5–5.65**	**3.9/4.8**	**92/94**

## Data Availability

Not applicable.
